# Supplementary material for: Auditory cortex shapes sound responses in the inferior colliculus
Source: eLife. 2020 Jan 31;9:e51890. doi: 10.7554/eLife.51890 (PMC7062464; doi:10.7554/eLife.51890)
Supplement: Transparent reporting form [file elife-51890-transrepform.docx]

***eLife’s* transparent reporting form**

We encourage authors to provide detailed information *within their submission* to facilitate the interpretation and replication of experiments. Authors can upload supporting documentation to indicate the use of appropriate reporting guidelines for health-related research (see [EQUATOR Network](http://www.equator-network.org/%20)), life science research (see the [BioSharing Information Resource](https://biosharing.org/)), or the [ARRIVE guidelines](http://www.plosbiology.org/article/info:doi/10.1371/journal.pbio.1000412) for reporting work involving animal research. Where applicable, authors should refer to any relevant reporting standards documents in this form.

If you have any questions, please consult our Journal Policies and/or contact us: [editorial@elifesciences.org](mailto:editorial@elifesciences.org).

**Sample-size estimation**

- You should state whether an appropriate sample size was computed when the study was being designed
- You should state the statistical method of sample size computation and any required assumptions
- If no explicit power analysis was used, you should describe how you decided what sample (replicate) size (number) to use

Please outline where this information can be found within the submission (e.g., sections or figure legends), or explain why this information doesn’t apply to your submission:

Methods - electrophysiological recordings: We used power analysis for effect size of 25% and expected variance in firing rate (50%) to determine the minimum total number of units for each experimental group at N=44.

**Replicates**

- You should report how often each experiment was performed
- You should include a definition of biological versus technical replication
- The data obtained should be provided and sufficient information should be provided to indicate the number of independent biological and/or technical replicates
- If you encountered any outliers, you should describe how these were handled
- Criteria for exclusion/inclusion of data should be clearly stated
- High-throughput sequence data should be uploaded before submission, with a private link for reviewers provided (these are available from both GEO and ArrayExpress)

Please outline where this information can be found within the submission (e.g., sections or figure legends), or explain why this information doesn’t apply to your submission:

Methods - electrophysiological recordings: For cohorts with awake and anesthetized recordings data were analyzed separately, but we observed no difference in our results so data were combined (Figure 8 – figure supplement 1). All groups included a greater number of units (single- and multi-units were combined in analysis). The number of units in measuring tone-evoked responses are specified in Table 1. Mice that did not show effect of laser activation or suppression in auditory cortex were excluded.

**Statistical reporting**

- Statistical analysis methods should be described and justified
- Raw data should be presented in figures whenever informative to do so (typically when N per group is less than 10)
- For each experiment, you should identify the statistical tests used, exact values of N, definitions of center, methods of multiple test correction, and dispersion and precision measures (e.g., mean, median, SD, SEM, confidence intervals; and, for the major substantive results, a measure of effect size (e.g., Pearson's r, Cohen's d)
- Report exact p-values wherever possible alongside the summary statistics and 95% confidence intervals. These should be reported for all key questions and not only when the p-value is less than 0.05.

Please outline where this information can be found within the submission (e.g., sections or figure legends), or explain why this information doesn’t apply to your submission:

**Methods: Statistical Analyses.**

Significant differences and *P* values were calculated using paired Wilcoxon sign-rank test (unless noted otherwise) with standard MATLAB routine. For the laser alone data, to compare distributions to standard normal distribution data were normalized by mean and standard deviation and then significant differences and P values were calculated by Kolmogorov-Smirnoff test with standard MATLAB routine. Mean ± standard error of the mean was reported unless stated otherwise. * indicates p < 0.05, ** indicates p < 0.01, *** indicates p < 0.001.

Statistics are reported in results and figure legends.

(For large datasets, or papers with a very large number of statistical tests, you may upload a single table file with tests, Ns, etc., with reference to sections in the manuscript.)

**Group allocation**

- Indicate how samples were allocated into experimental groups (in the case of clinical studies, please specify allocation to treatment method); if randomization was used, please also state if restricted randomization was applied
- Indicate if masking was used during group allocation, data collection and/or data analysis

Please outline where this information can be found within the submission (e.g., sections or figure legends), or explain why this information doesn’t apply to your submission:

Methods - STRF analysis: To calculate the STRF we separated the stimulus into 1-second chunks, concatenating the 250 ms laser ON chunks and the 250 ms laser OFF chunks immediately preceding laser onset. These data were then used to calculate the average spectrogram preceding a spike. To allow for finer temporal resolution of the STRFs we upsampled the DRCs using nearest neighbor interpolation. Subsequently we averaged the STRF across the eight stimulus files. To determine the significance of the lobes, the *z*-score of pixels was computed relative to the baseline values from an STRF generated with scrambled spike trains, using Stat4ci toolbox (Chauvin, Worsley, Schyns, Arguin, & Gosselin, 2005; Natan, Carruthers, Mwilambwe-Tshilobo, & Geffen, 2017). We ran this significance test 100 times and any pixel identified as significant more than 90 times was considered significant. Lobes were matched between laser ON and laser OFF trials by comparing the overlap of the lobes, requiring a 50% overlap of the smallest lobe size to be a match.

**Additional data files (“source data”)**

- We encourage you to upload relevant additional data files, such as numerical data that are represented as a graph in a figure, or as a summary table
- Where provided, these should be in the most useful format, and they can be uploaded as “Source data” files linked to a main figure or table
- Include model definition files including the full list of parameters used
- Include code used for data analysis (e.g., R, MatLab)
- Avoid stating that data files are “available upon request”

Please indicate the figures or tables for which source data files have been provided:

Original spike data and code are available on Dryad (<https://datadryad.org/review?doi=doi:10.5061/dryad.1t61c80>).
